# Malaria and the ‘last’ parasite: how can technology help?

**DOI:** 10.1186/s12936-018-2408-0

**Published:** 2018-07-11

**Authors:** Ngoc Minh Pham, Walter Karlen, Hans-Peter Beck, Emmanuel Delamarche

**Affiliations:** 1 Department of Health Sciences and Technology, ETH Zürich, Lengghalde 5, 8092 Zurich, Switzerland; 2 Swiss Tropical and Public Health Institute, Socinstrasse 57, 4051 Basel, Switzerland; 3 University of Basel, Petersgraben 1, 4001 Basel, Switzerland; 4 IBM Research-Zurich, Säumerstrasse 4, 8803 Rüschlikon, Switzerland

**Keywords:** Malaria, Rapid diagnostic tests, Elimination, Microfluidics, Smartphones

## Abstract

Malaria, together with HIV/AIDS, tuberculosis and hepatitis are the four most deadly infectious diseases globally. Progress in eliminating malaria has saved millions of lives, but also creates new challenges in detecting the ‘last parasite’. Effective and accurate detection of malaria infections, both in symptomatic and asymptomatic individuals are needed. In this review, the current progress in developing new diagnostic tools to fight malaria is presented. An ideal rapid test for malaria elimination is envisioned with examples to demonstrate how innovative technologies can assist the global defeat against this disease. Diagnostic gaps where technology can bring an impact to the elimination campaign for malaria are identified. Finally, how a combination of microfluidic-based technologies and smartphone-based read-outs could potentially represent the next generation of rapid diagnostic tests is discussed.

## The burden of malaria

The first record of malaria fevers dates back to the 5th century BC [1]. Today, malaria remains one of the four most life-threatening infectious diseases worldwide, together with tuberculosis, HIV/AIDS and hepatitis [2]. Latest data published by the World Health Organization (WHO) are staggering: more than 216 million cases in 91 countries and more than 400,000 deaths occurred globally in 2016 [3]. These figures are the same as in 2015, indicating that despite the unprecedented efforts in recent years, progress has stalled. This calls for more effective tools to reduce malaria and finally to eliminate this scourge. If this historical milestone can be accomplished, it could save the global economies $2 trillion by 2040 [4].

## Current diagnostic technologies and the challenges of detecting the ‘last’ parasite

This review only focuses on relevant innovative diagnostic technologies for malaria elimination settings where the malaria transmission is low; therefore, there is a critical need to detect asymptomatic individuals. Together with other effective interventions, ultra-sensitive rapid diagnostic tests are much needed to identify the invisible reservoirs. The role of innovative tools becomes crucial in the fight against malaria and the WHO identifies three strategic pillars (universal access to prevention, drugs and diagnosis, elimination and surveillance), of which accurate and effective diagnostics at the point-of-care (POC) is the first step towards appropriate diagnosis and treatment for malaria infection [5, 6].

Table 1 compares the performance of currently available malaria diagnostic tests for case management and surveillance. The landscape for malaria diagnosis can be divided into two main groups, POC methods in case management and laboratory-based methods for surveillance [7]. In case management, microscopy and RDTs are the two diagnostic methods that are recommended in primary settings whilst highly sensitive RDTs and molecular diagnostics [polymerase chain reaction (PCR) and loop mediated isothermal amplification (LAMP)] are often used in laboratory settings [8]. While presenting ultra-sensitivity (less than 2 parasites/μL for both Pan and P*f*-LAMP) in the field [9, 10], implementing malaria diagnostic tools in the field still requires addressing of several critical challenges such as simplified sample preparation steps, ready to use kits that require no cold chain [11]. Further, there is no reported literature referring to the use of malaria LAMP as a diagnostic tool in populations, or of being endorsed and procured by any programs or governments. In the meantime, also being less sensitive, conventional RDTs are at much lower cost of approximately 1 $USD per test [12]. Field studies have shown that POC methods such as microscopy and rapid diagnostic tests (RDTs) are effective in low-resource settings (LRS) [10, 13–25].

**Table 1:** Characteristics of current malaria diagnostic tools used in case management and surveillance

	LoD (p/µL or ng mL^−1^)	Sensitivity (%) (95% CI)	Specificity (%) (95% CI)	Cost ($US/test)		Time	Other requirements
				Instrument	Test		
Case management							
Microscopy	Expert: 4–20 [18]	Depends on microscopist		~ 3000	0.12–0.40 [19]	60 min [18]	Trained personnel, microscope, Giemsa stain [18]
	Average: 50–200 [19]						
RDTs	Existing RDTs: 100 p/µL [22] Latest product: 80 pg/mL for P*f*HRP2 [21]	> 85% depending on species [19]	> 99% [19]	No need for expensive instrument	0.55–1.50 [18]	20 min [20]	Test kit, appropriate storage conditions [18]
Surveillance							
RDTs	Latest product: 80 pg/mL for P*f*HRP2 [21]	> 85% depending on species [19]	> 99% [19]	No need for expensive instrument	0.55–1.50 [18]	20 min [20]	Test kit, appropriate storage conditions [18]
PCR	26 (real-time) [10]	100% [23]	> 99% [10]	Real-time instrument > 20,000 [25]	1.5–4.0 [24]	Standard > 6 h	Thermocycler, cold chain, power, reagent grade, water
	− 0.5 to 5. 0 [24]						
LAMP	47 (real-time) [10]	83.3% [22]	> 99% [22]	Conventional PCR and LAMP ~ 5000 [25]	0.40–0.70 [24]	60 min	Heat source for amplification and DNA extraction
	≥ 1 [23]	97.3% [24]	> 85% [23]				

*p/µL* parasites/µL, *LoD* limit of detection, *CI* confidence interval

## Microscopy

Microscopy is the reference standard for visualization of parasites in blood smears with an analytical sensitivity under normal circumstances approximately tenfold inferior than that of molecular testing [26]. Microscope has been commonly used as a diagnostic tool in peripheral health centres for various reasons, including availability [27]. However, the quality of such diagnosis depends on the availability and skills of trained microscopists, which might not always be available in the LRS, where malaria is endemic.

## Rapid diagnostic tests

Field studies have confirmed the benefits of introducing RDTs into routine testing such as better case management, improved adherence to test results, and having more rational treatments [28, 29]. Characteristics of current malaria RDTs are summarized in Table 2. Key advantages of RDTs are the ease to use and quick result delivery time (15–20 min). Unlike PCR or microscopy, RDTs detect circulating antigen; therefore they can also be used to detect placental malaria [30]. Diagnosis of malaria in pregnancy is challenging because of placental sequestration, which is specific to *Plasmodium falciparum* infections, can make microscopy detection of parasites difficult.

**Table 2:** Advantages and disadvantages of current malaria RDTs

Advantage	Disadvantages
Easy to use	Deletion of the P*f*hrp2 gene leads to false negative RDTs (particularly in populations in the Amazon region)
Low cost	Lack of adequate sensitivity for detection of infection in asymptomatic individuals and/or prozone effect
Quick result delivery time (< 20 min)	Lack of heat stability when being stored in endemic settings
Portable and disposable	Inability to differentiate non-P*f* malaria
Require minimal laboratory infrastructure, power or external equipment	Inability to distinguish current and past infections
Quick training	Inability to quantify parasite density, especially for assessing severity of illness or monitoring treatment efficacy

Although using the same technology of lateral flow immunoassays, the performance of malaria RDTs varies greatly from brand-to-brand, and lot-to-lot, especially with specimens having low parasite density (< 200 parasites/μL). In a collaboration between the Foundation for Innovative New Diagnostics (FIND), the WHO and the Centers for Disease Control and Prevention, 293 malaria RDTs were evaluated from 2008 to 2016 [31]. Most of the evaluated malaria RDTs detect *P. falciparum* histidine-rich protein 2 (P*f*HRP2) or *P. falciparum* lactase dehydrogenase (P*f*pLDH). In the last round of evaluation, anomalies that interfered with result interpretation were also recorded [31]. The most common anomalies were incomplete clearing and red background, which were observed in 48 and 24% of products. The second most common anomalies were failed migration of liquid, incomplete migration and patchy broken test lines, which occurred in 15, 11 and 11% of the products, respectively.

The performance of lateral flow-based RDTs depends on two main factors: the sensitivity and specificity of antibody-antigen combinations, and the ability to facilitate reliable liquid migration on the nitrocellulose membrane. Much research has focused on new biomarker discovery [32–34], and only limited attention has been paid to reduce limitations imposed by the inhomogeneous migration of liquid across porous nitrocellulose membranes [35].

**Fig. 1:**
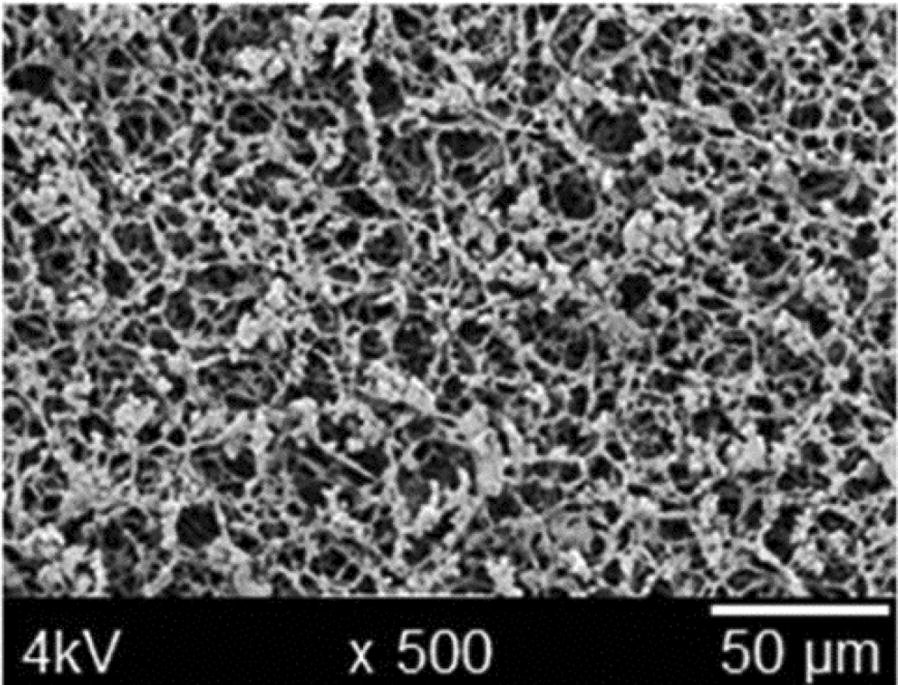
Scanning electron micrograph showing the porousity of nitrocellulose membrane (Reprinted with permission from [36] copyright 2014 Royal Society of Chemistry)

Figure 1 illustrates how unstructured the flow paths could be in a nitrocellulose membrane [36]. As the migration of liquid occurs in a porous network and is not actively controlled, a number of limitations arise: large volumes of sample needed, accumulation of reagents at the leading edge of the liquid flow, and increased cross-reactivity [37]. It is, therefore, time to consider alternative options to facilitate a more precise liquid migration, hence more accurate test results.

## Promising and alternative technologies for malaria detection

Table 3 summarizes six major classes of technologies used for detecting malaria and indicates their maturity levels. These technologies are individually reviewed in depth elsewhere [38] and most of them rely on standard concepts using immunoassays [39, 40], molecular diagnostics [41–49] and the visualization of parasites [50–53]. Table 4 provides specifications of some recently entered market malaria diagnostic [38]. Of those market-ready products, four of them are molecular diagnostics, three are immunoassays and one is based on automated microscopy. Several promising proof-of-concepts for the next generation of malaria RDTs are emerging. For example, prototypes have been built to detect the presence of haemozoin in blood sample [54–57]. Haemozoin crystals are produced by *Plasmodium* parasites as a final non-toxic compound of haemoglobin metabolism. In a specific example, a portable light meter was built to image crystalized haemozoin pigment [58]. These pigments are birefringent, so the detection of haemozoin is based on rotating a plane of polarized light through them and observing anisotropic output of the light. The minimum concentration of haemozoin that could be detected with this polarized light system was 15 pg/mL, equivalent to 30 parasites/μL of blood. Applications in the field are to be tested.

**Table 3:**
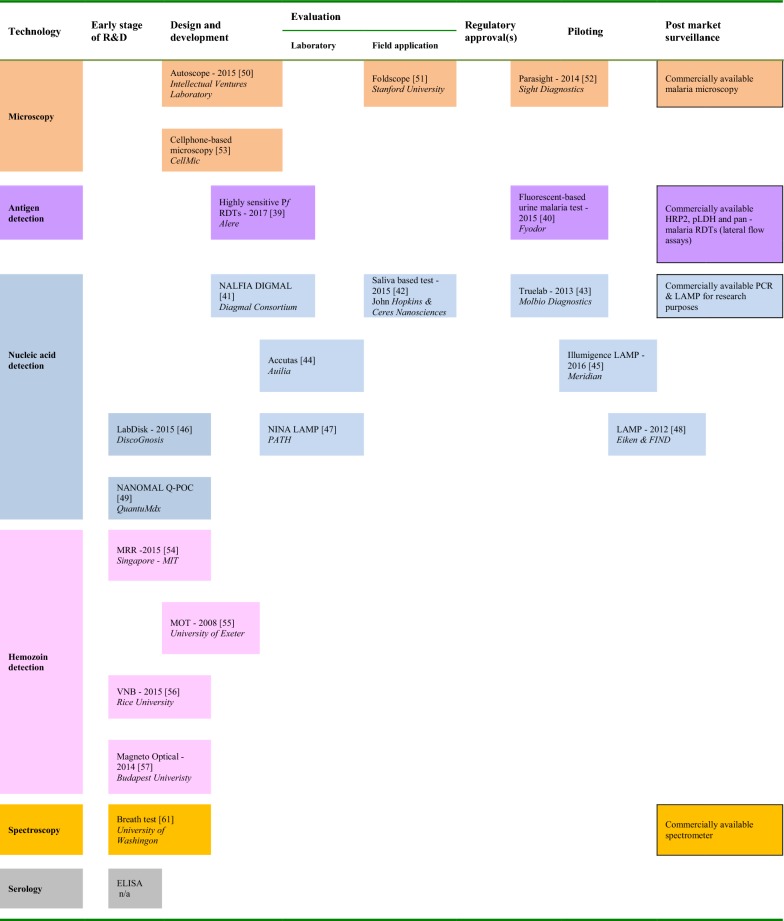
Examples of promising technologies for point-of-care diagnostics. table based on information contained in Ref [38]

*LAMP* loop-mediated isothermal amplification, *MRR* magnetic resonance relaxometry, *NINA* non-instrumented nucleic acid amplification, *MOT* magneto-optical technology, *VNB* Homozoin-generated vapour nanobubble

**Table 4:** Specifications of recently-entered market* technologies for malaria diagnosis. table based on information contained in Ref [38]

Technology	Product	Developer	Description	Type of detection	Performance	Turn-around time	Sample type	Environmental requirements	Cost per test	Cost per instrument	Power/labour/infrastructure requirements	Result display and storage	Quality control
Microscopy	Parasight	Sight Diagnostics Ltd, 2014	Automated microscopy suitable for processing of multiple malaria	Slide reading	Under way	n/a	Blood smear	n/a			n/a	n/a	n/a
Malaria RDTs**	Fio-net	Fio Corporation, 2012	Universal RDT reader and cloud information services to improve malaria RDT quality assurance and malaria surveillance	Combination of mobile diagnostics (mobile universal reader) with cloud information services	Automated and customising reports Sensitivity and specificity are functions of the RDTs being read	RDTs processing time is dependent on manufacturer’s recommendation Data upload within minutes Daily quality control needed	Depending on RDTs’ manufacturers	Subject to RDTs manufacturers’ recommendations 5–40 °C	Similar to pre-paid cellphone plans		Battery powered Basic 1 day training needed	On screen and web portal	CE marked
	UMT	Fyodor Biotechnologies, 2015	A senstitive and specific lateral flow assay detecting novel *Plasmodium* proteins shed in the urine of febril malaria patients	Dipstick technology (lateral flow assay)	LOD 125 parasites/µL	~ 20 min	100 µL urine	n/a	n/a		Usable by lay people	n/a	n/a
	Holomic Rapid Diagnostic Reader	Holomic LLC, 2013	Universal RDT reader attachment for smartphones and software to read RDTs and transmit result to a secure cloud information service	Portable, smartphone-based lateral flow immunoassay reader	Quantitative and qualitative	RDTs processing time is dependent on manufacturer’s recommendation Data upload within seconds	Depending on RDTs’ manufacturers	Subject to RDTs manufacturers’ 5–40 °C	Customisable	$US500	Battery powered Basic < 0.5 day training needed	User interface of the smartphones application	Class I medical device
Nucleic acid detection	LAMP Malaria Diagnostic Kit	Eiken Chemical Ltd and FIND, 2012	Commercial LAMP test kit containing primers and reagents needed to run assays using benchtop laboratory equipment	Isothermal DNA amplification Fluorescence of visual detection	For pan-LAMP: 97.0% sensitivity For Pf-LAMP: 93.3% sensitivity 85.0% specificity	60 min	30–60 µL blood	Stable for 12 months at < 30 °C	$US5	$US10’000	Electricity (batter-powered possible) 4 days of training required	Turbidimeter and software	CE marked Positive and negative controls included
	illumigene LAMP	Meridian Bioscience	An automated and compact LAMP technology to qualitatively detect *Plasmodium spp*. DNA in human whole blood samples	Isothermal DNA amplification	Sensitivity 100% Specificity 89.3%	< 50 min	Human whole blood	Stable for 12 months at 2–30 °C	n/a		Does not require specialised laboratory equipment	n/a	CE marked
	MicroPCR	Tulip Group and Bigtec Labs, 2013	POC real-time quantitative PCR instrument	Fluorescent probe-based real-time PCR	> 99% sensitivity and specificity LOD 2 parasites/µL blood	45–60 min	100 µL blood	15–30 °C	$US15	$US8000	Battery powered 1–2 days training required	5000 test results can be stored internally, cloud information available	CE marked
	Truelab	Molbio, 2013	A quantitative micro PCR platform containing all equipment and reagents needed for point-of-care applications	Using the proprietary magnetic nanoparticles to capture DNA	n/a	< 60 min	Whole blood	n/a			n/a	A customised micro printer is available	n/a

* Recently-entered market means products pass the regulatory and policy stage

** G6PD point-of-care tests are not included due to lack of information for popular products. CareStart G6PD RDT (AccessBiO) and POC G6PD (PATH) are working on promising products

Another example utilizes a portable breath analyzer: breaths of malaria-infected patients were found to contain terpenes, a family of aromatic chemicals that are produced by parasites that can further attract mosquitoes [59, 60]. A pilot study in Malawi confirmed that these aromatic compounds could be transported into the lungs and hence could be detected in the exhalation of infected patients [61].

Despite being unquestionably novel, these abovementioned methods of detection still need to prove their practicality for POC in LRS and demonstrate a clinically relevant limit of detection (LOD). For instance, in the breath analyzer, it would be useful to be able to convert the level of terpenes detected in breath into parasite density.

## Specifications for a new generation of malaria RDTs

Different settings require different target product profiles (TPP) [8]. Unlike previous malaria control campaigns, the key characteristics of malaria elimination efforts are to interrupt endemic transmission and to prevent its re-establishment [62]. The Program for Appropriate Technology in Health (known as PATH) and FIND are pioneering the development and validation of sensitive rapid tests for mass screening in LRS. They also proposed a TPP for malaria RDTs in elimination settings, stating specific requirements for the ideal rapid tests according to concept of Affordable, Sensitive, Specific, User-friendly, Equipment-free and Deliverable (ASSURED) [63]. The desired LOD is 5 parasites/µL or less, or concentration range of 6–12 ng/mL P*f*HRP2 [63]. For RDT developers it is important to note the caveat of the prozone phenomenon that might prevent detection of high parasite density [64]. Poor specificity could lead to over-treatment, thus depreciation of the intended value of RDTs (from public health perspectives); therefore, the required specificity for effective malaria diagnosis is at least 97% or ideally 99% [63].

Additional requirements for ideal RDTs are suitability and appropriateness for LRS where most malaria cases occur. To make an impact simplicity and affordability are of utmost importance. Simplicity means, the system should be equipment-free and should require very little resources [65]. A simple and automated test could obviate false results caused by user-errors [66]. Affordability is difficult to measure and depends on the cost–benefit equation of a specific situation. Also, tests should be designed to minimize impact of inappropriate storage conditions (2–40 °C) on reagent stability and usability of the devices [67].

## Microfluidic technology for malaria POC testing

**Fig. 2:**
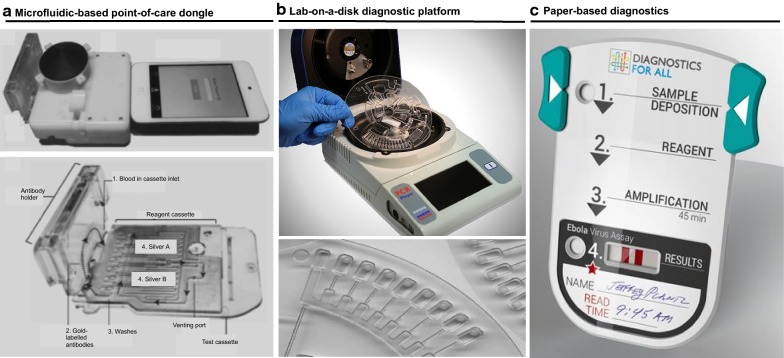
Examples of microfluidic-based diagnostics for low resource settings. Reprinted with permission: **a** from [72], copyright 2015 The American Association for the Advancement of Science, **b** from [73], copyright 2017 Royal Society of Chemistry, **c** from [74] copyright 2018, Diagnostics for All. Image courtesy of Diagnostics for All

Microfluidics enable the miniaturization and simplification of complicated analytical processes while consuming less reagents, minimizing waste, and requiring less supporting instrumentation [68]. This stems out from the predictable behaviour of liquids at the microscale where flow is typically laminar. At microscale, minute amounts of liquids can be manipulated using microstructures, such as microvalves, micromixers or micropumps [69]. Low volumes of reagents, fast reaction times, compact and portable platforms are just a few advantages that make microfluidics technology attractive for POC applications [70, 71]. Figure 2 shows several examples demonstrating the archetype of microfluidic-based diagnostics for POC applications, which is an integrated system composed of a disposable unit (where analysis takes place) and a signal acquisition and processing module to process the results. (a) [72], (b) [73], (c) [74].

Currently, microfluidic-based diagnostic devices can be divided into two categories: non-paper-based “traditional” microfluidics and paper-based microfluidics [75, 76]. Research on traditional microfluidics often focuses on miniaturizing conventional techniques. For example, a collection of passive and active mixing elements were designed to facilitate mixing processes on chips [77]. Recent work in developing microfluidic-based diagnostic devices has focused on integrating all necessary elements into stand-alone platforms [78, 79] because such integrated systems can operate without bulky accessories and do not require water, buffer, or a constant supply of electricity [80]. There are many ways to control liquid flows on microfluidic platforms, for instance, acoustic forces, mechanical forces, magnetic forces, as well as capillary and centrifugal forces [81–85]. To satisfy the stringent requirements for LRS, devices based on capillary and centrifugal forces have shown promising results. Table 5 presents some examples of microfluidic-based systems that have been designed to detect P*f*HRP2 and P*f*pLDH antigens or genetic materials from the parasites using on-chip molecular testing, cell deformation mechanism, electrical, optical, and magnetic detections amongst others [54, 58, 79, 81, 86–94].

**Table 5:** Performance of proof-of-concept platforms based on microfluidics for malaria detection

Application	Concept/detection principle	Biomarker/target	Limit of detection	Performance		Time (min)	Refs
				Sensitivity (%)	Specificity (%)		
Molecular analysis	Paper-based LAMP	*P. falciparum*	5 p/µL	61%	98%	45 min	[81]
		*P. vivax*		81%	98%		
		*P. pan*		> 80%	> 98%		
	Continuous flow PCR	*P. falciparum*	2 p/µL	97.40%	93.80%	n/a	[86]
			< 1 p/µL	n/a	n/a	2.5 h	[87]
Cell deformation mechanism	Inertial focusing	*P. falciparum*	2–10 p/µL	n/a	n/a	400 µL/min	[88]
	Inertial microfluidics	*P. falciparum* iRBCs	2 cells/min	n/a			[89]
	Non-inertial lift effect	*P. falciparum* ring stage iRBCs	Enrichment factor of 4.3	n/a			[90]
			Throughput 12,000 cells/h				
Electrical detection	Electrical conductivity of iRBCs is significantly higher than healthy RBCs	*P. falciparum* ring stage	n/a	n/a			[91]
	Optofluidic-flow analyser that can measure the optical absorption of RBCs in P. *falciparum* infected blood sample	*P. falciparum*	1712 RBCs/s	n/a		3 min	[92]
			2.96% parasite density				
	Naked-eye screening of in-meso detection of hemozoin crystallites based on birefringence	Hemozoin crystals produced by *P. falciparum*	n/a			~ 12 min	[58]
Optical detection	Visual detection of colored assay spot on a disposable microfluidic card based on a flow-through membrane immunoassay	Malaria P*f*HRP2	10–20 ng/mL	n/a		1–5 min	[79]
	Paper-based catridge containing detection areas for both thin and thick smears	*P. falciparum*	100 p/µL	n/a		30 min	[93]
Magnetic detection	Cell enrichment microfluidics combined with magnetic relaxometry detection	*P. falciparum* ring stage parasites	5% parasite density	n/a		15 min	[54]
	Detection of hemozoin in iRBCs by magnetic resonance relaxometry	Hemozoin in iRBCs in *P. falciparum* infections	< 10 p/µL	n/a		Few mins	[94]

*RBC* red blood cell, *iRBC* infected red blood cell

## Immunodiagnostics on microfluidic platforms for malaria detection

**Fig. 3:**
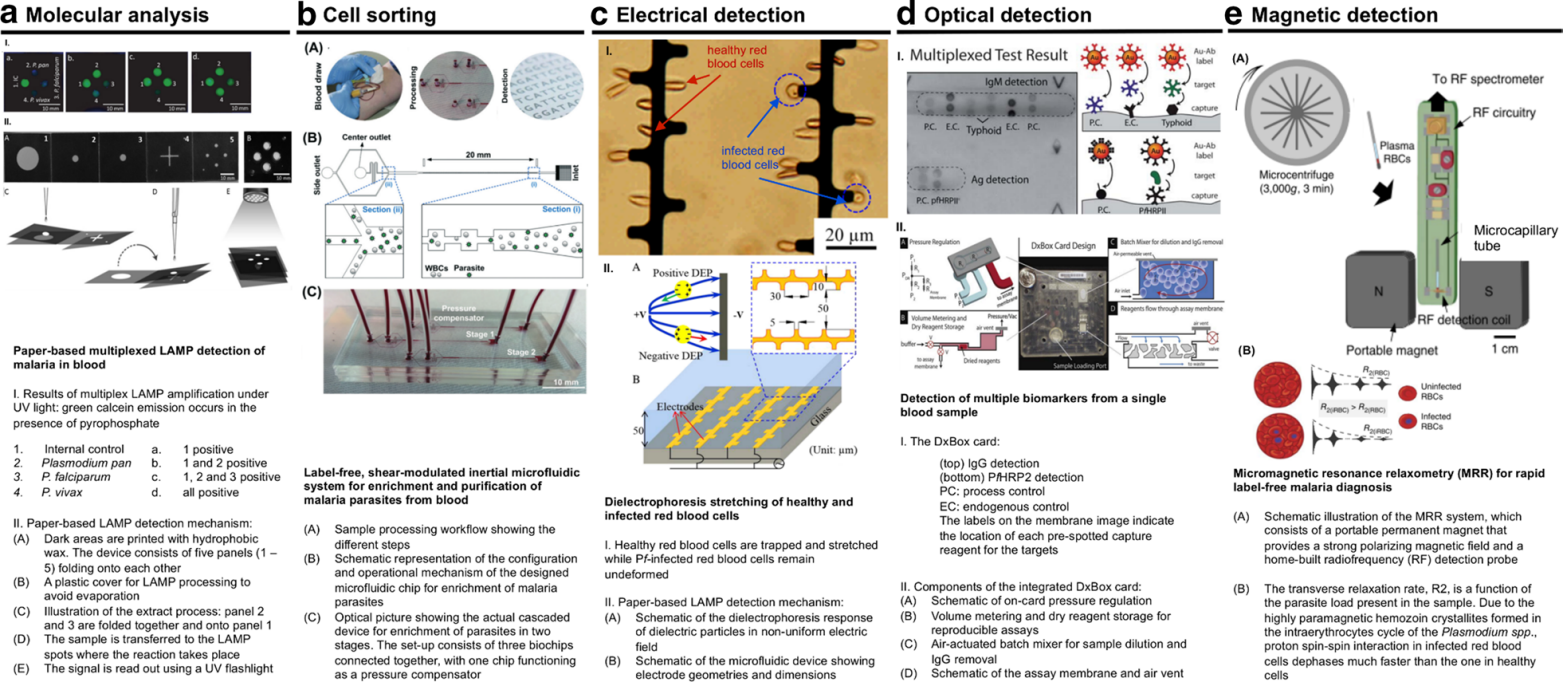
Examples of microfluidic prototypes for malaria diagnosis using different methods. Reprinted with permission: **a** from [86], copyright 2016 Wiley–VCH, **b** from [77], copyright 2014 Royal Society of Chemistry, **c** from [80] copyright 2014 Elsevier, **d** from [68] copyright 2012 Royal Society of Chemistry, **e** from [83] copyright 2014 Springer Nature

Standard protocols to perform immunodiagnostics on microfabricated platforms require sample pre-concentration, flow control and detection of biomarkers (analytes and/or parasites). These multi-step protocols can benefit greatly from miniaturization, and in fact, microfluidic-based immunoassays have demonstrated their potential for reliable and accurate performance [95, 96]. Figure 3 presents some examples to illustrate how microfluidics technology can be used to detect malaria by different methods of detection, such as molecular testing, size-based cell sorting, electrical differentiation of healthy and infected red blood cells, optical detection of antigen and magnetic detection of haemozoin. (a) [97], (b) [88], (c) [91], (d) [79], (e) [94].

### Sample pre-concentration

Low antigen concentration is a common problem in diagnostic immunoassays and malaria antigen detection is not an exception. To overcome this challenge, several prototypes of analyte concentrator have been developed to enrich biomarkers hence improve LOD. To illustrate how analyte enrichment prior to analysis can improve sensitivity of ELISA, Cheow et al. reported a prototype that can enhance the LOD of prostate-specific-antigen assay up to 1.85 pg/mL [98]. The significant enhancement of 100-fold was achieved by trapping the charged fluorescent product of standard ELISA (analyte-bound enzyme complex) using a multiplex electrokinetic preconcentration technique without modifying the immunobinding process.

Blood is the most common type of specimen for POC testing. However, the cellular components in whole blood often cause non-specific background. To address this problem, a continuous microfluidic device was developed to filter the cells, making plasma available for on-chip analysis [99].

Healthy and *P. falciparum*-infected red blood cells exhibit different ionic permeability of their plasma membrane, with infected cells being more permeable. Therefore, when healthy and infected cells are suspended in a low conductivity medium, infected cells lose internal ions and acquire a different dielectrophoretic mobility than healthy ones [100]. Several groups have developed microfluidic chips using dielectrophoresis and variants of it to separate cells successfully leading to promising prototypes for detecting infected red blood cells thus malaria infections [101–103].

### Flow control

Controlling flow on microfabricated devices often introduces a great degree of complexity. For example, a combination of screws, pneumatic and solenoid valves was integrated into a microfluidic platform to actuate flow and control chemical gradients in microchannels [104]. This design might be suitable for laboratory-based tests, but may not lead to robust systems for LRS. Nonetheless, the uses of centrifugation and capillary forces to transport liquids are excellent examples of stand-alone systems [105, 106]. Extensive reviews discussing how to engineer flow path in microscale using capillary and centrifugal forces for POC applications exist [69, 107]. Libraries of microfluidic elements such as valves, mixers and pumps have also been developed [77, 108, 109].

### Detection

Sensitive detection remains one of the biggest hurdles for clinical diagnosis at the onset of infection. The bottleneck is the limited amount of detectable analytes in a very limited volume of sample. One strategy is to amplify the signal, then convert it into quantitative measurements such as electrical and/or optical signals [96]. The detection strategy is therefore critical for the overall design and fabrication of a device. Optical detection is considered as the ideal read-out for POC applications of microfluidics owing to the simple design and potentially low cost [110, 111]. There are five main categories of optical detection based on the type of generated optical signals: fluorescence, luminescence, absorbance, surface plasmon resonance, and surface-enhanced Raman scattering [112–116]. Detailed discussions about detection strategies for microfluidics systems also exist in the literature [117].

## Molecular testing on microfluidic platforms for malaria detection

At the moment, PCR and LAMP are the most sensitive technique for identification of asymptomatic individuals, for example, in 130 clinical samples presenting no parasites based on microscopy, as low as 3.6 × 10^−4^ parasite/μL could be identified in 117 samples by a highly sensitive genus-specific quantitative reverse transcriptase real-time PCR (qPCR) [118]. This low LOD was achieved by amplifying and detecting the total nucleic acids of the 18S rRNA genes, which increased the analytical sensitivity of the assay by more than 1 log unit compared to DNA only. However, current applications of PCR and LAMP are still restricted to well-equipped laboratories and thus not suitable for LRS [119]. Miniaturized PCR and/or LAMP is desirable, but developing such devices is a more challenging task than that for biomarkers detection for three reasons: (1) sample pre-treatment is essential for extracting DNA of parasites for downstream analysis, (2) the critical signal amplification step highly depends on temperature control, and (3) robust, low cost, and portable detection techniques are required for remote settings [120].

### Sample pre-treatment

The PCR/LAMP process requires isolation of genetic materials from infected cells, pre-concentration, as well as signal amplification and analysis. All steps need to be integrated seamlessly in a closed process to overcome time consuming laboratory-like processing steps. Earlier studies have demonstrated successful prototypes that could sequentially perform cell isolation and lysis for messenger RNA purification [121]. On this device, a unique valving system was designed to facilitate liquid migration and analysis. Microfluidics with “macrofluidics” can also be combined to precisely reconstitute reagents, and automated filling liquids for multiplex PCR technique. A successful story is the Cepheid GeneXpert instrument, where all steps from sample preparation, nucleic acid extraction, to thermal cycling for amplification and eventually detection can be integrated into one platform [122]. A review of microfluidic-based DNA analysis systems is available here [123].

### Heating systems

The major challenge of miniaturizing bench-top PCR instruments is the requirement of numerous heating cycles for thermal reactions. To overcome this challenge, micromixers and microchambers were designed to allow thermal reactions to take place rapidly [124]. To speed up DNA amplification by improving thermal transfer through interfaces, microfluidic elements, such as mixers, heaters and temperature controlling units were integrated into glass and silicon substrates [125]. Another strategy to enable different heating regions using continuous flow was investigated using a Peltier element to regulate the temperature for thermal cycling [86]. On this platform, as few as to 2 *P. falciparum* parasites/μL could be detected. This device offered a simplified sample processing step using desiccated hydrogel, reagents and a camera to detect amplicons. When analysing 188 archived, frozen samples collected in Uganda, this prototype achieved 97.4% sensitivity and 93.8% specificity.

One of the most promising development for stand-alone integrated systems for DNA analysis perhaps was an elegant combination of an exothermic reaction with phase change materials to regulate the heat for thermal cycling [126]. In this prototype, downstream processes such as purification and concentration of sample were integrated seamlessly into the same platform.

Recent work reported by Juul et al. challenged the need of thermal cycling for PCR-like systems by proposing an endogenous enzyme activity detection called rolling-circle enhanced enzyme activity to quantify as little as 1 *P. falciparum* parasite/μL [87]. The principle of this method is based on using rolling-circle-amplification (RCA) technique to convert a circular DNA template into a 10^3^ tandem repeat rolling-circle product. In this system, RCA substrates can be processed by the DNA-cleaving enzyme topoisomerase I from *Plasmodium* parasites, which produces many DNA circles leading to enhanced signal. RCA products can have sizes reaching micrometers, which enable visualization at single molecular level.

## Paper-based microfluidics

Paper-based microfluidics was proposed by Whitesides and colleagues [127]. Since then, this technology has been growing fast with great promises for global health applications [128]. Unlike its sister products of paper test strips, paper-based microfluidic analytical devices offer well-defined, millimetre-sized microchannels to transport liquids in a controlled manner, yet with low cost for production (< $0.01) [129]. Using hydrophobic “inks” to define areas on hydrophilic paper, it is possible to perform multiple immunodiagnostic assays on the same test strip. To illustrate how complex analytical processes can be simplified and transformed into a paper-based microfluidic device, Pereira et al. integrated concentration and detection steps into a single step assay [130]. The analyte P*f*pLDH in low abundance was first accumulated using a micellar aqueous two-phase system (ATPS). The micellar ATPS consisted in a nonionic Triton X-114 surfactant, which was used to concentrate biomarkers in a sample and enhance the LOD. In this system, a tenfold improved LOD of 10 ng/μL P*f*pLDH was achieved. In an alternative development of a foldable, card-like test device, P*f*HRP2 could be detected and quantified [131]. The generated signal in presence of P*f*HRP2 was amplified by gold nanoparticles, yielding a LOD of 1.2 ng/mL P*f*HRP2, which is four times higher than that of the unamplified case. These studies serves as excellent examples for low cost, non-instrumented analysis systems without compromised performance. Many other innovative approaches to control liquid flows such as selective hydrophobic rendering or origami in which folding of multiple paper layers to trigger reactions were also investigated successfully [132–134].

## Interfacing microfluidic-based analysis with networked mobile devices

Mobile health applications have rapidly been growing in recent years and there is a trend in interfacing consumer electronics such as smartphones with lateral flow RDTs or microfluidic-based devices [135, 136]. Such combination is expected to deliver increased objectivity of test result interpretation and improved connectivity of the entire healthcare systems. The automation and digitized test results can be more easily combined with other health related parameters and combined with medical decision support systems. User-friendly interfaces, automated result analyses, remote-monitoring and data aggregation, increased storage conditions, and active quality assurance are just a few additional benefits of this approach [137].

In 2008, paper-based microfluidics were integrated with a smartphone camera to perform immunoassays [128]. The camera of the phone was used to take a photograph of the detection zone before and after the deposition of specimen. Since then, many groups have started to develop and enhance capabilities of phone-based low cost diagnostic readers [136]. Table 6 presents an overview of recent work in developing phone-based prototypes that can be used to detect variety of biomarkers for a wide range of diseases with clinically relevant performance. Devices are designed for a broad spectrum of applications, from genetic testing, cancer detection to personalized food allergen monitoring [136, 138–140]. A wide range of strategies are also derived to enhance signal strength, for instance, using Quantum dots, Rayleigh/Mie scatter or gold nanoparticles [141–143]. At present, applications of smartphone-based diagnostics for malaria detection can be divided into two categories: phone-based RDT readers, which provides automatic interpretation of results, and phone-based brightfield microscopes, which allow simple and portable means to visualize parasites in blood samples [138–149].

**Table 6:** Examples of lab-on-a-phone applications

Optical detection	Data analysis	Signal transduction	Target biomarker	Sample	Platform	Performance	Refs.
Phone LED and camera + 4 external lenses and mirrors	Mie scattering simulation online	Immunoagglutination (Mie light scattering)	P*f*HRP malaria biomarker	Human blood	Microbeads	1 pg/mL–10 ng/mL	[144]
						LOD 1 pg/mL	
Computational power + external optical fiber + LED	Phone application	Fluorescence	Genomic DNA	*Escherichia coli* and *Staphylococus aureus*	Microfluidics	Comparable to that of commercial PCR	[138]
Phone camera	Phone app	Colorimetry	HE4 (ovarian cancer biomarker)	Urine	Microchip	89.5% sensitivity, 90% specificity	[139]
2 external LEDs + phone camera	Phone app	Colorimetry	Peanut	Cookies	Sample holder	< 1 ppm	[140]
External LED + phone camera + additional lens	Phone application	Fluorescence	*Escherichia coli*	Milk, water	Glass capillary	5–10 cfu/mL	[141]
External LED and optical fibers	Phone app	Immunochromatography (Mie scatter)	Thyroid stimulating hormone	Human serum	Nitrocellulose test strip	0.31 mIU/L	[142]
Phone camera + external LED	Computer	Colorimetry	Human IgG	Human IgG sample	Microfluidics, silver deposition	n/a	[143]
Snap-on attachment (lens + LEDs) + phone camera	Phone app	Immunochromatography	Malaria biomarkers	Whole blood	Rapid test diagnostic strips	4 × dilution c.f. RDTs	[145]
3 external attachments + lenses + LED + phone camera	Phone application	Fluorescence	Cell count	Blood	Sample holder	600–2500 white cells/image	[146]
						400–700 red cells/image	
Phone camera	Phone app	Colorimetry	pH		Test strip	n/a	[147]
External LEDs and photodiode	Phone app	Colorimetry	Glucose	Urine	Paper strips	0–250 mg/dL	[148]
						LOD 10 mg/dL	
Snap-on attachments (lens + LED) + phone camera	ImageJ on computer	Fluorescence	Prostate specific antigen (PSA)	Whole blood	Microfluidics	Dynamic range 0.08–60 ng/mL	[149]
						LOD 0.4–0.04 ng/mL	

## Phone-based RDT readers

A smartphone was used for quantitative reading of the Optimal-IT test, a commercially available malaria RDT with a snap-on unit as reader that is suitable for both Android and iPhone [145]. Images of RDTs were acquired, in either transmission or reflection, and then processed in real time to deliver test results within 10 min. The spatio-temporal information collected by this device can document prevalence of many infectious diseases and would allow efficient tracking of epidemics. Another approach to integrate a custom microfluidic-based immunoassay detecting P*f*HPR2 with phone-based detection was the development of a microfluidic chip, which can be connected to a phone camera to analyze signals and deliver results in 10 min. The opto-mechanical unit in this case consisted of optical fibers, microfluidic chips and mirrors, and could be easily removed from the back camera of the phone. The principle was to quantify changes in fluorescent intensity upon capturing of P*f*HPR2 on the sensing region, yielding a LOD of 1 pg/mL of P*f*HRP2 in 10% diluted blood [144].

## Phone-based bright-field microscope

Accurate and consistent blood smear reading is challenging to attain in health centres or small clinics in remote regions. A phone-based microscope is a low cost option that can offers enhanced image quality, improved accuracy and user comfort [146, 150]. There are two simplified imaging techniques suitable for smartphone apps: (1) lens-free holographic imaging, and (2) on-lens devices.

Holography is an image-constructing technique using scattering and interference of light and pixel super-resolution to enhance optical images [151]. An automated lens-less holography was developed with a sufficient field of view of 24 mm^2^ to visualize and capture images of *P. falciparum* in blood smears [152].

Phone-based microscopy can also be engineered to be a field-ready polarized light microscope without compromised fidelity and resolution [153]. The principle was to detect light birefringence caused by the crystallization of haemozoin. This field-based, modular microscope could magnify *Plasmodium chabaudi* parasites up to 50 times, gaining a comparable performance compared to conventional polarized microscope. Additional benefits of this prototype are simple operations and low cost per test. Further work using clinical samples could confirm the full potential of this novel phone-based polarized light microscope.

## Conclusion

Accurate and effective diagnosis is the first step to further pursue efforts to eliminate and reduce the global burden of malaria by 90% in 2030. Current diagnostic methods can detect malaria symptomatic infections, but often miss out asymptomatic cases. The rise in proportion of asymptomatic infections in low transmission areas calls for a new generation of rapid diagnostic tests that can detect the hidden parasite reservoir. Technology is advanced nowadays to (at least theoretically) be able to track down the last parasite carriers. While malaria case management has improved, other causes of fever need to be detected and treated accordingly. Therefore, the ideal RDT should come in as a complete package with ultra-high sensitivity and specificity, meet the ASSURED standards for LRS, and also provide additional diagnostic capabilities. Microfluidic devices coupled to phone-based readouts offer a unique opportunity to not only reduce the burden of infectious diseases, such as malaria, but also could provide tools for monitoring epidemics and elimination progress on very large scales.
